# Serotype and molecular diversity of nasopharyngeal *Streptococcus pneumoniae* isolates from children before and after vaccination with the ten-valent pneumococcal conjugate vaccine (PCV10) in Ethiopia

**DOI:** 10.1186/s12879-019-4024-1

**Published:** 2019-05-10

**Authors:** Wondewosen Tsegaye Sime, Abraham Aseffa, Yimtubezenash Woldeamanuel, Sarah Brovall, Eva Morfeldt, Birgitta Henriques-Normark

**Affiliations:** 1 Armauer Hansen Research Institute, Jimma Road, 1005 Addis Ababa, Ethiopia; 2 Department of Microbiology, Parasitology and Immunology, Saint Paul’s Hospital Millennium Medical College, 1271, Addis Ababa, Ethiopia; 3 Department of Microbiology, Immunology and Parasitology, Addis Ababa University, College of Medicine and Health Science, 9086, Addis Ababa, Ethiopia; 4 The Public Health Agency of Sweden, Stockholm, Sweden; 5 Department of Microbiology, Tumor and Cell Biology, MTC, Karolinska Institutet, 171 77 Stockholm, Sweden; 6 Clinical Microbiology, Karolinska University Hospital, 171 76 Stockholm, Sweden

**Keywords:** *Streptococcus pneumoniae*, Carriage, Serotype, Vaccination, PCV, Infants, Ethiopia

## Abstract

**Background:**

*Streptococcus pneumoniae* is a major human pathogen, and nasopharyngeal colonization is the first step for transmission and pathogenesis of pneumococcal diseases. Ethiopia introduced the 10-valent pneumococcal conjugate vaccine (PCV10) in October 2011. Here we studied nasopharyngeal carriage rates of pneumococci in children and analyzed the serotype and genetic diversity of pneumococcal isolates before first dose and after completion of the vaccine.

**Method:**

A longitudinal study was conducted from February 2013 to November 2016. Totally 789 infants were enrolled at the age of 6 weeks before first dose of PCV10 vaccination, 206 were re-sampled at the age of 9 months, and 201 at 2 years of age after the final dose of PCV10 at the age of 14 weeks. One hundred sixteen children were followed during all the three sampling periods. A total of 422 nasopharyngeal isolates were serotyped using gel diffusion and the Quellung reaction, 325 were typed with pulsed field gel electrophoresis (PFGE), and 12 were selected for multi locus sequence typing (MLST).

**Results:**

Pneumococcal carriage rates at the age of 6 weeks, 9 months and 2 years of age were 26.6% (210/789), 56.8% (117/206) and 48.3% (97/201), respectively. Out of 116 children none of them carried the same strain during the three period and the carriage rate at the age of 6 weeks, 9 months and 2 years were 32.7% (38/116), 59.% (69/116) and 49.1% (57/116) respectively. Totally 59 pneumococcal serotypes were identified among 422 isolates. Serotype 6A (5.0%) dominated followed by 34 (4.5%), 10A (4.0%), 11A (4.0%), 19F (3.8%), 15B (3.8%), 23F (3.6%), and 15A (3.6%). The proportion of non-PCV10 serotypes among the isolates recovered at 6 weeks, 9 months and 2 years was 79.4, 88.9 and 89.7% respectively. Molecular typing of 325 isolates collected at 6 weeks and 9 months of age showed a high genetic diversity.

**Conclusion:**

This study highlights the presence of very diverse serotypes in Ethiopia where non-vaccine serotypes were predominant. Completion of the PCV10 schedule was associated with an approximately 50% reduction of vaccine-type carriage and increase of non-vaccine types. PCV13 would potentially reduce vaccine-type carriage by further 10%.

**Electronic supplementary material:**

The online version of this article (10.1186/s12879-019-4024-1) contains supplementary material, which is available to authorized users.

## Background

*Streptococcus pneumoniae* (the pneumococcus) is among the top human pathogen with high rates of morbidity and mortality, with one of the largest public health concern and economic impact of any bacterial infectious agent in both developing and industrialized countries [1–6]. It is reported that each year, approximately half of the 2.6 million deaths due to acute respiratory infections in under five year children are caused by pneumococcal pneumonia, the majority occurring in the developing countries [3, 7]. It has also been estimated that in 2015 pneumococcal disease caused about 294, 000 deaths in children aged 1–59 months [8].

Pneumococci are the major cause of common infections such as community-acquired pneumonia, otitis media, sinusitis, and also a major contributor to severe invasive infections such as septicaemia and meningitis. The nasopharynx of children is the ecological niche for pneumococci and nasopharyngeal colonization is a prerequisite for pneumococcal diseases. The polysaccharide capsule is a major pneumococcal virulence factor and more than 97 pneumococcal capsular serotypes have been identified with different potential to cause invasive pneumococcal disease (IPD) [9–13]. The serotype distribution in pneumococcal diseases and carriage varies with time, geographical areas and age of the population. While some serotypes are found frequently in either or both disease and carriage, others are rarely isolated [14]. Currently available pneumococcal vaccines are based on the pneumococcal capsule, either as a polysaccharide based vaccine targeting 23 serotypes (PPV23) or as conjugated vaccines targeting a limited number of serotypes, 7 in PCV7 (serotype 4, 6B, 9 V, 14, 18C, 19F and 23F), 10 in PCV10 PV7 plus serotypes 1, 5 and 7F) and 13 in PCV13 (PCV10 plus serotypes 3, 6A and 19A). Analysis of the serotype distribution in IPD and carriage as well as the disease incidence in a particular area is important to evaluate the effectiveness of the vaccines [6, 15–17] .

A number of studies have shown that incidences of both invasive and non-invasive pneumococcal diseases have declined after PCV introduction in vaccinated children, and in some countries they also observed a herd protection effect in non-vaccinated populations such as the adults [18–20] . However, other countries have not observed any herd protection effects on IPD in non-vaccinated elderly due to an almost complete replacement of vaccine types with non-vaccine types, both in IPD and in carriage in small children [21, 22]. A reduction in pneumococcal carriage of PCV7 serotypes and an increase in colonization by non-PCV7 serotypes was observed after introduction of PCV7 in year 2000 in the United states [23]. From year 2010, PCV7 was changed to either PCV10 or PCV13 in European countries and in the United States [24–27] .

Ethiopia introduced PCV10 in the routine vaccination program in October 2011 to be given at the age of 6 weeks, 10 weeks and 14 weeks (3 + 0) without catch-up vaccination for older children. A few studies have reported nasopharyngeal carriage rates and found rates ranging from 41 to 78% [28–30], but there is still a need for adequate baseline information on epidemiological factors such as the rate of carriage and transmission, serotypes and genetic relatedness of isolates, for subsequent impact assessment in Ethiopia. The objective of this study was to determine the carriage rates and analyze the phenotypic and genetic diversity of pneumococcal isolates in children before the first dose and after completion of PCV10 vaccination in the post-vaccination era.

## Methods

### Study population and study site

This study was conducted in Addis Ababa city that had an estimated population of 3.35 million during the study period (February 2013 to November 2016), of which 1.76 million were female [31]. There were more than 100 public and private health facilities providing the PCV10 vaccine when the study was started and the average number of children vaccinated since the introduction of the vaccine in October 2011 was in the range of 22,000–23,000 children per month. According to the Ethiopian federal ministry of health report of 2016, the PCV10 vaccine coverage reached 100% in Addis Ababa and 97.5% at overall national level in 2015 [32].

The study was conducted in seven health centres selected randomly from a total of 36 heath centres from seven different sub-cities. The required sample size was computed using the general formula for a single population proportion with the following assumptions: prevalence rate of 79% taken from Abdullahi et al.*,* 2012, Kenya [33], 95% confidence level and 3% marginal error. Considering 10% for anticipated non-response rate, the total sample size calculated was 789. Infants were recruited sequentially when they came for the first dose of PCV10 at age 6 weeks. The sample size for follow up was calculated based on the actual finding of 6 weeks carriage rates, with the power of 80% to observe 50% change in vaccine type serotypes carriage after completion of the vaccine. For follow-up, 206 participants at 9 months and 201 at the age of 2 years were re-sampled, and 116 children were sampled during all the three periods. All the children who participated in the study didn’t attend the day care centre until their two-years of age.

Infants with any signs of respiratory infection or antibiotic exposure within 2 weeks at the time of sampling were excluded from the study.

### Sample collection and processing

Samples and data were collected by nurses working in the vaccination unit at each health centre. Nasopharyngeal swab specimens were collected following standard operating procedures at each period [34]. A flexible calcium alginate-tipped swab (Fisher Scientific, UK) was used. The swab tip was transported in a cryotube containing 1 ml skim-milk-tryptone-glucose-glycerin medium (STGG) [34, 35] to the bacteriology laboratory of the Armauer Hansen Research Institute (AHRI). Each swab was plated within 6 h of collection on trypticase soy agar (TSA) plates supplemented with 5% sheep blood containing 5 μg/ml gentamicin (Fisher Scientific, UK). All plates were incubated at 37 °C for 18–24 h in a 5% CO_2_ atmosphere.

### Iidentification of *S. pneumoniae*

Colonies on TSA plates with macroscopic appearance characteristic for pneumococci (small, greyish, and colonies demonstrating α-hemolysis) and Gram-positive diplococci were sub-cultured on TSA to get single pure colonies. Then suspect α-hemolytic pneumococcal colonies were characterized based on optochin (5 μg ethylhydrocupreine) (Fisher Scientific, UK) sensitivity and bile solubility [34, 36]. Confirmed pneumococcal isolates were stored in duplicate in 1.5 ml STGG broth cryotubes at -80 °C until transported to Karolinska Institutet, Sweden, for serotyping and molecular characterization.

### Serotyping

Serotyping was done at the Public Health Agency of Sweden using gel diffusion and/or the Quellung reactions using 46 type or group sera, and isolates belonging to a group that included more than one type such as serogroups 6, 7, 9, 10, 11, and others were examined by the capsular reaction test with type-specific factor antisera [37]. Of 424 *S. pneumoniae* isolates identified, 422 isolates were available for serotyping. The antisera used were purchased from Statens Serum Institute in Copenhagen, Denmark.

### Pulsed field gel electrophoresis (PFGE)

PFGE analysis was carried out for 325 isolates collected from children at the age of 6 weeks and 9 months. *S. pneumoniae* DNA embedded in agarose blocks (Bio-Rad, USA) was prepared from log phase bacterial culture as previously described [38, 39]. The blocks containing genomic DNA were digested with *Apa* I (Promega, USA) restriction enzyme at 25 °C overnight. PFGE was performed by the contour-clamped homogeneous electric field method on a CHEF DR-III apparatus (Bio-Rad Laboratories, Inc., USA) in 0.5x Tris-Borate-Ethylene diamine tetraacetic acid (TBE) buffer (pH 8.0) in 1.2% agarose gel for with ramped pulse times from 2 to 30 s for 22 h at 6 V/cm at 14 °C [40].

The DNA banding profiles were stained with GelRed™ (Biotium, USA) and by the Gel Doc 1000 documentation system. Conversion, normalization and further analysis of patterns were carried out with the Gel Compare software version 4. The level of similarity between the PFGE patterns was calculated using the Dice coefficient, and correlation coefficient was calculated with the UPGMA (unweighted pair group methods with arithmetic averages) as previously described [41]. An isolate was considered to be within a cluster if the band matching tolerance was 1%, optimization 1% and the coefficient of similarity was 80%.

### Multi locus sequence typing (MLST)

Twelve pneumococcal isolates collected from children at 6 weeks and 9 months of age representing 11 PFGE genotypes were analyzed by MLST, as described previously [42], and according to the MLST website (http://spneumoniae mlst.net/). The resulting allelic profiles were concatenated to confirm pneumococcal identity [43] and analyzed to determine sequence type. Isolates that differ at one of the seven loci of STs included in the MLST database were defined as single locus variant (SLV) of the defined ST. Correlation was assessed between the sequence types (ST) obtained and all existing STs in the database, using the eBURST (electronic Based upon Related Sequence Type) software available at the MLST website (http://pubmlst.org/spneumoniae).

### Statistical analysis

All statistical analyses were performed using Statistical Package for Social Sciences (SPSS) version 20.0 statistical software (IBM corporation, Chicago, IL, USA) and STATA version 11(Statacorp, College Station, TX). Carriage rates for the three sampling periods were calculated with 95% confidence intervals (95%CI). The proportions of carriage and serotype variations were computed within the three sampling period using t-test, and the Pearson’s Chi-square statistical analysis was applied to determine variation in difference in carriage rate among health centres. All statistical tests were considered significant at *p*-values< 0.05.

## Results

### Nasopharyngeal pneumococcal carriage rates were highest at 9 months

The carriage rates among the children recruited in the health centers ranged from 20 to 33% before the children were vaccinated at the age of 6 weeks. There was no statistically significant variation in proportion of carriage rate (*p* = 0.113) among the health centers (Table 1). The second round of nasopharyngeal samples were taken from 206 children at the age of 9 months from those children who participated in the first sampling at the age of 6 weeks. The total carriage rate in 9 months old children, all fully vaccinated with PCV10, was 56.8% and the carriage rate at health centre level ranged from 48.1 to 65.2%. As was observed in the first round of samples, there was no statistically significant variation in the carriage rate among the health centres (*p* = 0.112). Also at the age of two years there was no statistically significant difference in the carriage rate among health centres (*p* = 0.897) and the carriage rate ranged from 36.4 to 62.5% (Table 1).

**Table 1:** Nasopharyngeal carriage rate of *S. pneumoniae* at the age of 6 weeks before the first dose of the vaccine and 9 months and 2 years after completion of PCV10 vaccination: distribution by site of recruitment

Health Centers	At the age of 6 weeks			At the age of 9 months			At the age of 2 years		
	Total sample	Positive	Percent (95% CI)	Total sample	Positive	Percent (95% CI)	Total sample	Positive	Percent (95% CI)
Addis Ketema	117	39	33.3 (24.9–42.6)	47	34	65.2 (57.4–84.4)	39	22	56.4 (39.6–72.2)
Arada	59	16	27.1 (16.4–40.3)	–	–	–	–	–	–
Beletshachew	102	21	20.6 (13.2–29.7)	20	10	50.0 (27.2–72.8)	32	20	62.5 (43.2–78.9)
Bole-17	110	31	28.2 (20.0–37.6)	45	23	51.1 (34.7–65.4)	38	22	57.9 (40.1–73.7)
Dil Fire	141	38	27.0 (19.8–35.1)	44	22	50.0 (34.6–65.4)	34	12	35.3 (19.7–53.5)
Kirkos	140	28	20.0 (13.7–27.6)	27	13	48.1 (28.7–68.0)	47	17	36.2 (22.8–51.5)
Kolfe	120	37	30.8 (22.7–39.9)	23	15	65.2 (42.7–83.6)	11	4	36.4 (10.9–69.2)
Total	789	210	26.6 (23.6–29.8)	206	117	56.8 (49.7–63.6)	201	97	48.3 (40.2–54.4)

Among children sampled during all three periods (*n* = 116) the nasopharyngeal carriage rates of *S. pneumoniae* was the highest at the age of 9 months (59.4%), and non-vaccine types dominated (94.2%) (Table 2). Only 15 out of the 116 children (12.9%) were carriers throughout the three periods. Importantly, we did not identify any child carrying the same serotype persistently at the age of 6 weeks, 9 months and 2 years .

**Table 2:** Nasopharyngeal carriage rates and pneumococcal serotype distribution in the 116 children sampled during all three study time points

Age of sampling	Pneumococcal carriage n (%)	PCV10 vaccine types n (%)	Serotypes (n)
Age of 6 weeks	38 (32.7)	7 (18.4%)	6B(1), 19F(1), 23F(4), 9 V(1)
Age of 9 months	69 (59.4)	4 (5.8%)	6B(1), 19F(2), 23F(1)
Age of 2 years	57 (49.1)	4 (7.1%)	19F(3), 23F(1)

### Longitudinal follow up of PCV 10 vaccinated children

**Fig. 1:**
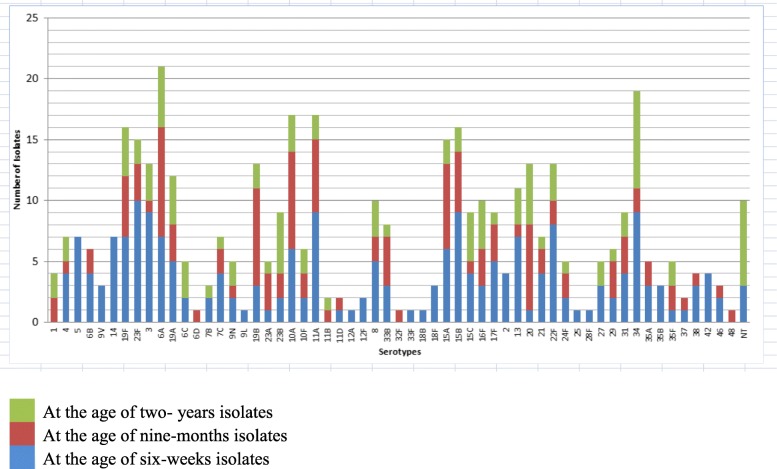
Serotype Distribution of *S. pneumoniae Isolates* from Infants at the initiation (6 weeks) and after completion of PCV10, at 9 months and at 2 years of age

From the totally 422 isolates, 59 different serotypes were identified, and 10 isolates were non-typeable (NT). Fifty-four different serotypes were found among the 208 children before vaccination (6 weeks of age), 43 different serotypes among the 117 isolates from children at 9 months of age, and 37 different serotypes among the 97 at the age of 2 years old post-vaccination isolates (Fig. 1). Serotypes 6A (5.0%), 34 (4.5%), 10A (4.0%), 11A (4.0%), 19F (3.8%), 15B (3.8%), 23F (3.6%), and 15A (3.6%) dominated in decreasing order, covering nearly 32% of all isolates. Only three of the predominant serotypes (19F, 23F and 6A) are included in currently available PCVs. Total vaccine coverage for the three conjugated vaccines were 13.7% for PCV7, 15.4% for PCV10 and 26.3% for PCV13 among all isolates, hence non-vaccine types (NVT) dominated (84.6% for PCV10 and 73.7% for PCV13). Among colonized children, vaccine serotypes 5, 14, 23F and 19F occurred more frequently before (14.9%) than after immunization (6.8%) (*p* = 0.031). Serotypes 6A, 34 and 10A were major serotypes after vaccination found in 16.4% compared with 10.1% before the vaccine was given (*p* = 0.067).

Among serotypes included in PCV10, 13 isolates of serotypes 1, 4, 6B, 19F, and 23F from the age of 9 months, and 10 isolates of serotypes 1, 4, 19F and 23F from the age of 2 years were identified in the samples collected after vaccination. Among non-vaccine types, one third of the isolates were belonging to serotype 34 (4.5%), 11A (4.0%%), 10A (4.0%), 15B (3.8%), 15A (3.6%), 19B (3.1%) and 22F (3.1%) in decreasing frequency. Approximately 11% (46/422) were serotype 3, 6A and 19A, which are not included in PCV10, but present in PCV13 (Fig. 1).

Among 117 carriers of pneumococci at the age of 9 months, 44 (37.6%) also carried *S. pneumoniae* at the age of 6 weeks and only 4 (9.1%) children had the same serotype at both time points. These serotypes were 6A in two children and 23F and 33B in two other children, while the remaining 40 (90.9%) were colonized by different serotypes. Seventy-three (62.4%) of the children that were colonized at the age of 9 months did not carry pneumococci at the age of 6 weeks (Additional file 1). Out of the 97 children who carried *S. pneumoniae* at the age of 2 years, 29 (29.9%) were also positive at the age of 6 weeks and all children were colonized with different serotypes (Additional file 2). Among the 57 children that were colonized with pneumococci at the age of 2 years, 37 (64.9%) carried pneumococci both at the age of 9 months and at 2 years of age, and only three children carried the same serotypes (Additional file 3) .

**Fig. 2:**
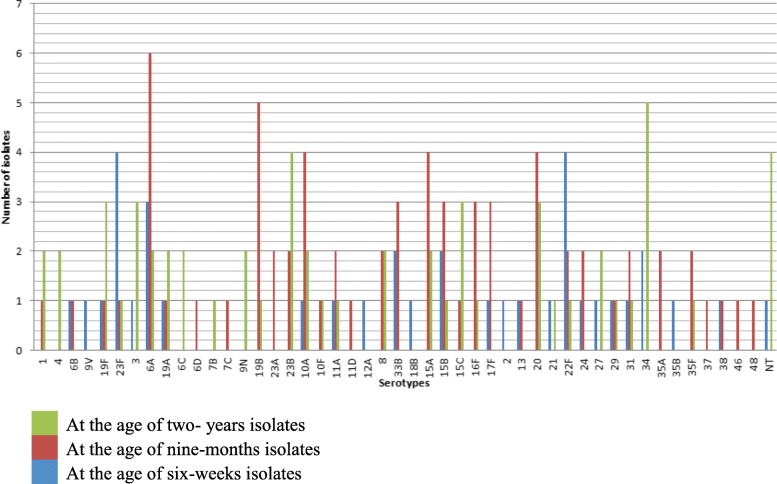
Serotype distribution of *S. pneumoniae* isolates from the same children at the age of 6 weeks, 9 months and 2 years

Among the 116 children that were sampled at all the three sampling points, 47 different serotypes were identified and serotypes 19F and 23F, both included in PCV10, were detected after full vaccination, both at 9 months and at 2 years of age. Similarly, serotype 6B was present at the age of 9 months and serotype 4 at the age of 2 years. Serotypes 19F, 23F, 6A, 19A, 10A, 11A, 15B, 22F, 29, and 31 were detected at all the three period. Non-vaccine types dominated during all the three sampling periods (Fig. 2).

### Decline in vaccine type serotypes carriage

Out of the 208 isolates collected from 6 weeks old children before the first dose of the vaccine was given, 42 (20.2%) were of vaccine types and 166 (79.8%) were of non-vaccine types. After completion of vaccination at the age of 9 months, the percentage of vaccine serotypes was significantly reduced from 20.2% (42/208) to 11.1% (13/117) (*p* = 0.035). Similarly, at the age of 2 years, vaccine types were significantly less frequent compared to at the age of 6 weeks (*p* = 0.034) (Table 3). Out of all 97 children at the age of 2 years who carried *S. pneumoniae* in their nasopharynx, 10 (10.3%) had vaccine types and among the vaccine types 4 isolates were of serotype 19F.

**Table 3:** Comparison of serotypes among nasopharyngeal pneumococcal isolates collected before vaccination with PCV10 at the age of 6 weeks and after completion of vaccination at the age of 9 months and two years

Period of sampling	Vaccine type			Non-vaccine type		
	Total Isolates	%	95% CI	Total isolates	%	95% CI
At the age of 6 weeks	42	20.2	15.0–26.3	166	79.8	73.7–85.0
At the age of 9 months	13	11.1	6.0–18.2	104	88.9	81.7–93.9
At the age of 2 years	10	10.3	5.0–18.1	87	89.7	81.9–94.9

### The genetic diversity was high among the pneumococcal carriage isolates

A total of 325 isolates (208 from infants at 6 weeks and 117 from infants at 9 months of age) were analyzed using Pulsed field gel electrophoresis (PFGE). Among these, 23 isolates were indigestible by the restriction enzyme *ApaI*. Only eleven PFGE genotypes were identified that comprised three or more isolates per PFGE genotype (*n* = 50). The remaining 252 PFGE genotypes contained only one isolate each suggesting a high genetic diversity. Out of 11 PFGE genotypes, six PFGE genotypes consisted of one serotype and the PFGE genotype was named A to F. The other five PFGE genotypes contained a combination of serotypes and were named G to K (Table 4).

**Table 4:** PFGE genotypes among pneumococcal isolates collected from infants at 6 weeks and 9 months of age

PFGE Genotype	Total number of isolates	Serotype and number (n) of isolates	Time of isolation		Number of sites where strains of the same PFGE was isolated	Number of other isolates with the same serotype, not belonging to the genotype
			^a^Before vaccine(n)	^b^After vaccine(n)		
A	4	31 (4)	2	2	3	3
B	3	46 (3)	2	1	3	-
C	5	8 (5)	3	2	4	2
D	7	3 (7)	7	-	3	3
E	4	6A (4)	4	-	2	12
F	3	14 (3)	3	-	3	4
G	6	10A (4)	2	2	4	10
		5 (1),	1	-	1	6
		6A (1)	1	-	1	-
H	5	16F (3)	1	2	3	3
		7B (1),	1	-	1	1
		33B (1)	1	-	1	2
I	7	13 (2)	1	1	2	6
		12A (1)	1	-	1	-
		1 (1)	-	1	1	1
		20 (1)	1	-	1	7
		42 (1)	1	-	1	3
		23F (1)	1	-	1	12
J	3	6A (2)	-	2	1	-
		10A (1)	-	1	1	-
K	3	23F (1)	1	-	1	-
		18F (1)	1	-	1	2
		13 (1)	1	-	1	-

^a^Before taking the first dose of the vaccine

^b^After taken the last dose of the vaccine at the age of 14 weeks

Totally 12 isolates, two isolates from PFGE genotype I and one isolate each from the rest of the 10 PFGE genotypes, were subjected to Multi locus sequence typing (MLST). As depicted in Table 5 most of the sequence types (STs) identified were present in the available MLST database. However SLV489 (serotype 31), SLV8930 (serotype 23F) and SLV3460 (serotype 6A) were different from the already reported STs in one allele, allele (*gki*), allele (*spi*) and allele (*xpiI*), respectively. The two strains of serotypes 1 and 23F, belonging to PFGE genotype I, showed the same SLV 8930, even though they belonged to different serotypes, indicating capsular switch. The two 6A isolates that were of two different PFGE genotypes showed the same ST.

**Table 5:** MLST analysis of selected *S. pneumoniae* isolates

PFGE genotype	Time of isolation	Serotype of the MLST typed isolates	Sequence type
A	Before vaccine	31	SLV6489
H	Before vaccine	16F	ST6882
B	After vaccine	46	ST6450
C	After vaccine	8	ST3500
I	After vaccine	1	SLV8930
	Before vaccine	23F	SLV8930
D	Before vaccine	3	ST458
E	Before vaccine	6A	ST3460
F	Before vaccine	14	ST63
K	Before vaccine	23F	ST988
J	After vaccine	6A	SLV3460

Before vaccine: It means before the children are given the first dose of the vaccine; After vaccine: It means after completion of the full dose of the

## Discussion

Nasopharyngeal carriage is a prerequisite for pneumococcal transmission and invasive disease. Pneumococcal colonization starts during early infancy and the median age at first acquisition of carriage was reported to be 33 days of age in The Gambia [44], 38.5 days in Kenya [45], 8 weeks in Bangladesh [46] and 45.5 days in Thailand-Myanmar [47]. In our study, we found a carriage rate of 26.6% at the age of 6 weeks when the infants came for the first dose of the PCV10 vaccine. This early exposure in infancy is likely to play an important role in the development of infection at an early age.

Nasopharyngeal carriage rates of pneumococci have been shown to vary depending on geographic region and population [48]. In African and Asian countries, pneumococcal carriage rates were often reported to be high. In Africa, these included carriage rates of nearly 90% in The Gambia in children [44], and other reports from the continent range from 18.6% to as high as 93.4% [49–52]. Our finding of carriage rates, in fully vaccinated children at 9 months 56.8% and at 2 years 47.3%, is rather consistent with the high rate of carriage reported from most African countries and few studies done in Ethiopia [28–30]. This suggests that vaccine has no impact on the overall carriage rate.

Carriage studies can be used to predict the potential impact and effectiveness of different formulations of PCVs [53]. To our knowledge, this is the first study determining carriage rates of pneumococci and analyzing the impact of PCV10 immunization on pneumococcal carriage rates in Ethiopia using a longitudinal approach. Pneumococcal carriage rates increased significantly from 26.6% at the age of 6 weeks to 56.8% at the age of 9 months. Then carriage rates declined slightly to 48.3% when the children reached the age of 2 years. Similar situations have been reported from other studies in Malawi [54], Kenya [55] and The Gambia [44]. A number of studies have shown that introduction of PCVs into routine vaccination programs did not affect overall pneumococcal carriage rates, but rather commonly led to non-vaccine type replacement [25, 27, 55–57]. Hence, the overall carriage rate might not be affected by vaccination, while the serotype distribution might change, and this seems to have been the case in our study as well.

We identified 54 different serotypes among the at the age of 6 weeks isolates before the first dose of the vaccine, and about 80% of the isolates were of non-vaccine types, showing a highly diverse serotype distribution, which is in agreement with a report before vaccine introduction [28]. This highlights the presence of a sizable pool of strains in Ethiopia with the potential for replacement of vaccine type strains with non-vaccine types in invasive and non-invasive diseases. This will probably have an important impact on the effectiveness of vaccination.

Out of 117 isolates from 9 months old children who completed the vaccination, we identified 43 different serotypes of which 89% were non-vaccine types. Vaccine types decreased from 20.2% before vaccination to 11.1% after use of the vaccine at the age of 9 months. Similarly, out of 97 isolates from children at 2 years, 90% were of non-vaccine types, and we did not find a reduction of vaccine types in this age group, when we compared to the age of 9 months. Our finding is in agreement with the Kenyan study where the carriage rate of PCV10 vaccine serotypes were significantly reduced from 34% before the immunization to 13% after completion of vaccination at two years [58].

Several strains that belonged to PCV10 serotypes were detected in vaccinated children. These included 13 strains of serotypes 1, 4, 6B, 19F, 23F isolated from children at the age of nine months, and ten strains that belonged to serotypes 1, 4, 19F and 23F detected at the age of 2 years. The proportion of PCV10 serotypes such as 23F, 14 and 5 was higher before vaccination, and we did not detect any serotype 5, 14 and 9 V at the age of 9 months and 2 years. We did not identify any PCV10 serotypes 7F or 18C strains in our collection, but we detected related non-vaccine types 7C and 18F, serotypes that have been identified in clinical invasive specimens (CSF, blood, ear discharge, throat, pleural fluid, and sputum) from Gondar and Addis Ababa [59, 60]. Why children continue to be colonized with vaccine types after completion of the vaccination schedule is unknown and could be due to several reasons. One possibility is that the concentration of antibodies produced as a result of carriage may not be sufficient to prevent colonization. Higher concentrations of IgG might be required to avoid colonisation than those necessary to prevent IPD [61, 62].

Among the non-vaccine types found in our study, 13% were of serotypes 9 N, 20, 11A, 8, 16F, and these serotypes were previously reported to cause meningitis in children in Tikur Anbassa Hospital, Ethiopia [60]. In another study in Ethiopia, serotypes 8, 10A, 13, 20, 22A, 27 and 15C were isolated from clinical samples [59]. We detected all these serotypes in the nasopharynx of children except for serotype 22A. Serotype 10A was detected at a higher frequency than the other serotypes.

Among the infants that carried pneumococci at the age of 6 weeks (*n* = 210), 37.6% were still carriers at the age of 9 months. However, at 9 months (*n* = 117), only 4 children were still colonized with the same serotype as that at the age of 6 weeks. The serotypes were 6A in two children and 23F and 33B in the other children. PFGE analysis confirmed that these at the age of 9 months isolates were different from those obtained at 6 weeks of age. This finding indicates that there is a natural fluctuation in carriage rates of different serotypes rather than a persistence of the first colonizer. Presence of the same serotype at two different time points does not necessarily mean that the same strain will continue colonizing. Colonization by different members of the same serotype replacing each other over time is therefore possible.

The proportion and distribution of circulating serotypes not included in currently available PCVs, as well as their level of invasiveness, will be key determinants of the overall impact of the vaccine on disease. From countries that currently use PCV10 or PCV13, there are reports showing that non-vaccine serotypes such as 10A, 12F, 15A, 15B, 15C, 22F, 24F, 33F, 35B and 38 cause IPD [22, 26, 63, 64]. Serotypes 12F, 22F, 24F and 33F have been identified also to have high invasive disease potentials [26, 63–66]. All these non-vaccine types were identified in our study, and serotype 6A which is not included in PCV10, but part of PCV13, was a predominant serotype.

There are certain limitations with our study. The investigation was started one year and three months after the introduction of the vaccine and therefore, the first baseline data in our study might not be free of the effect of serotype replacement and herd protection. Also, only 116 children were sampled three times due to logistic problems. Moreover, due to high costs for MLST, we could only analyse 12 representative isolates from at the age of 6 weeks and 9 months isolates, selected based on the PFGE results. Furthermore, only a single colony was taken from each positive sample for serotyping, and hence we might have missed multiple serotypes in our analyses.

## Conclusions

We found a high diversity of pneumococcal serotypes in carriage. At the last sampling at 2 years of age a majority (86.3%) were of non-vaccine types, suggesting that the effectiveness of PCV10 vaccination might be hampered. However, already at 6 weeks at the first sampling almost 80% of the isolates were of non-vaccine types, indicating that the PCV10 vaccine will not target the majority of the serotypes carried in this age group. Molecular typing using PFGE and MLST revealed a high diversity and the existence of capsular switching events. Furthermore, even though several children were colonized repeatedly during the study period, no child was colonized with the same strain during the two-year period. This suggests that re-colonization is due to acquisition of new strains. The data presented will be of importance for deciding on future vaccination strategies in Ethiopia.

## Additional files

Additional file 1: **Table S6**. Nasopharyngeal carriage of *S. pneumoniae* serotypes in children at the age of 9 months (vaccinated) compared to type and frequency at the age of 6 weeks (pre-vaccination). (DOCX 15 kb)

Additional file 2: **Table S7.** Nasopharyngeal carriage of *S. pneumoniae* serotypes in children at the age of 2 years (vaccinated) (*n* = 201) compared to type and frequency at the age of 6 weeks (pre-vaccination). (DOCX 15 kb)

Additional file 3: **Table S8**. Nasopharyngeal carriage of *S. pneumoniae* serotypes in children at the age of 2 years (*n* = 116) compared to type and frequency at the age of 9 months. (DOCX 15 kb)

## Abbreviations

IPD: Invasive pneumococcal disease
MLST: Multi locus sequence typing
PCV: Pneumococcal conjugate vaccine
PFGE: Pulsed field gel electrophoresis
SLV: Single locus variant
ST: Sequence types
