# Supplementary material for: Serotype and molecular diversity of nasopharyngeal Streptococcus pneumoniae isolates from children before and after vaccination with the ten-valent pneumococcal conjugate vaccine (PCV10) in Ethiopia
Source: BMC Infect Dis. 2019 May 10;19:409. doi: 10.1186/s12879-019-4024-1 (PMC6511162; doi:10.1186/s12879-019-4024-1)
Supplement: Supplementary file 1 — Table S6. Nasopharyngeal carriage of S. pneumoniae serotypes in children at the age of 9 months (vaccinated) compared to type and frequency at the age of 6 weeks (pre-vaccination). (DOCX 15 kb) [file 12879_2019_4024_MOESM1_ESM.docx]

**Additional file 1.**

**Table S6. Nasopharyngeal carriage of *S. pneumoniae* serotypes in children at the age of 9 months (vaccinated) compared to type and frequency at the age of 6 weeks (pre-vaccination).**

| **Carriage status** | **Pneumococcal carriers at the age of 9 months** | | **Pneumococcal serotypes** | | | | **No pneumococci at the age of 9 months** | |
| --- | --- | --- | --- | --- | --- | --- | --- | --- |
|  | ­­­  No. | % | **Same** | | **Different** | | No. | % |
|  |  |  | No. | % | No. | % |  |  |
| Pneumococcal carrier at the age of 6 weeks | 44 | 37.6 | 4* | 9.1 | 40 | 90.9 | 24 | 27 |
| No pneumococci at the age of 6 weeks | 73 | 62.4 | - | - | 73 | - | 65 | 73 |
| **Total** | **117** | **100** | **4** |  | **113** |  | **89** |  |

Serotype: *6A(2), 23F(1), 33B(1), PFGE showed that the strains are not the same.
