# Supplementary material for: Serotype and molecular diversity of nasopharyngeal Streptococcus pneumoniae isolates from children before and after vaccination with the ten-valent pneumococcal conjugate vaccine (PCV10) in Ethiopia
Source: BMC Infect Dis. 2019 May 10;19:409. doi: 10.1186/s12879-019-4024-1 (PMC6511162; doi:10.1186/s12879-019-4024-1)
Supplement: Supplementary file 2 — Table S7. Nasopharyngeal carriage of S. pneumoniae serotypes in children at the age of 2 years (vaccinated) (n = 201) compared to type and frequency at the age of 6 weeks (pre-vaccination). (DOCX 15 kb) [file 12879_2019_4024_MOESM2_ESM.docx]

**Additional file 2 .**

**Table S7. Nasopharyngeal carriage of *S. pneumoniae* serotypes in children at the age of 2 years (vaccinated) (n=201) compared to type and frequency at the age of 6 weeks (pre-vaccination).**

| **Carriage status** | **Pneumococcal carriers at the age of 2 years** | | **Pneumococcal Serotypes** | | | | **No pneumococci at the age of 2 years** | |
| --- | --- | --- | --- | --- | --- | --- | --- | --- |
|  | ­­­  No. | % | **Same** | | **Different** | | No. | % |
|  |  |  | No. | % | No. | % |  |  |
| ­­­Pneumococcal carrier at the age of 6 weeks | 29 | 29.9 | - | - | 29 | 100 | 23 | 27 |
| No pneumococci at the age of 6 weeks | 68 | 70.1 | - | - | 68 | - | 81 | 73 |
| **Total** | **97** | **100** | **-** | **-** | **97** |  | **104** |  |
