# Supplementary material for: Serotype and molecular diversity of nasopharyngeal Streptococcus pneumoniae isolates from children before and after vaccination with the ten-valent pneumococcal conjugate vaccine (PCV10) in Ethiopia
Source: BMC Infect Dis. 2019 May 10;19:409. doi: 10.1186/s12879-019-4024-1 (PMC6511162; doi:10.1186/s12879-019-4024-1)
Supplement: Supplementary file 3 — Table S8. Nasopharyngeal carriage of S. pneumoniae serotypes in children at the age of 2 years (n = 116) compared to type and frequency at the age of 9 months. (DOCX 15 kb) [file 12879_2019_4024_MOESM3_ESM.docx]

**Additional file 3**

**Table S8. Nasopharyngeal carriage of *S. pneumoniae* serotypes in children at the age of 2 years (n=116) compared to type and frequency at the age of 9 months.**

| **Carriage status** | **­­­Pneumococcal carriers at the age of 2 years** | | **Pneumococcal Serotypes** | | | | **No pneumococci at the age of 2 years** | |
| --- | --- | --- | --- | --- | --- | --- | --- | --- |
|  | ­­­  No. | % | **Same** | | **Different** | | No. | % |
|  |  |  | No. | % | No. | % |  |  |
| ­­­Pneumococcal carrier at the age of 9 months | 37 | 64.9 | 3* | 8.1 | 34 | 90.9 | 32 | 27 |
| No pneumococci at the age of 9 months | 20 | 35.1 | - | - | 20 | - | 27 | 73 |
| **Total** | **57** | **100** | **3** | **-** | **54** |  | **59** |  |

* serotype 19F, 8, and 23B, PFGE analysis was not done for two-year isolates
